# Relationship between Oral Health Status and Bone Mineral Density in Community-Dwelling Elderly Individuals: A Cross-Sectional Study

**DOI:** 10.3390/healthcare9040432

**Published:** 2021-04-07

**Authors:** Mayu Takeda, Yuhei Matsuda, Kumi Ikebuchi, Miwako Takeda, Takafumi Abe, Kazumichi Tominaga, Minoru Isomura, Toru Nabika, Takahiro Kanno

**Affiliations:** 1 Oral Care Center, Department of Oral and Maxillofacial Surgery, Faculty of Medicine, Shimane University, Izumo 693-8501, Japan; mtakeda@med.shimane-u.ac.jp (M.T.); yuhei@med.shimane-u.ac.jp (Y.M.); ikebuchi@med.shimane-u.ac.jp (K.I.); 2 Center for Community-Based Health Research and Education (CoHRE), Organization for the Promotion of Project Research, Shimane University, Izumo 693-8501, Japan; cohre1@med.shimane-u.ac.jp (M.T.); t-abe@med.shimane-u.ac.jp (T.A.); 3 Tominaga Dental Office, Ohchi 696-0313, Japan; shika-t@ohtv.ne.jp; 4 Faculty of Human Science, Shimane University, Matsue 690-8504, Japan; isomura@hmn.shimane-u.ac.jp; 5 Department of Functional Pathology, School of Medicine, Shimane University, Izumo 693-8501, Japan; nabika@med.shimane-u.ac.jp

**Keywords:** number of remaining teeth, bone mineral density, community-dwelling elderly, cross-sectional study, propensity score analysis

## Abstract

The relationship between oral health status and bone mineral density has been poorly elucidated. We conducted a cross-sectional study to examine the relationship between oral health status and bone mineral density with data from healthy community-dwelling elderly individuals in Ohnan-cho, Shimane Japan who were recruited in 2019 for the Shimane Center for Community-Based Health Research and Education (CoHRE) study. The study included 702 participants (306 men and 396 women). The median age, bone mineral density, and number of remaining teeth were 69.0 years, 86.2%, and 26.0, respectively. The two groups (Low teeth group and High teeth group) showed significant differences in age, hemoglobin A1c (HbA1c) level, and masticatory function in men (*p* < 0.05). In women, age, number of untreated teeth, and masticatory function were significantly different (*p* < 0.05). The odds ratio of propensity score analysis for the association between the number of remaining teeth and bone mineral density was 27.7 (95% confidence interval: 1.86–414.9, *p* < 0.05). The number of remaining teeth could be associated with bone mineral density in the healthy elderly women, and no significant association was observed in men. Number of remaining teeth and bone mineral density may be interrelated, and oral care by dentists/dental hygienists may play an important role in maintaining bone mineral density in elderly women.

## 1. Introduction

In Japan, the population is aging more rapidly than in any other country in the world because of the world’s highest life expectancy and persistently low birth rate [1]. The population growth has shown a downward trend since 2008, but according to projections, by 2035, the number of individuals aged 65 years and above will reach 37.82 million, accounting for 33% of the total population, and the proportion is expected to increase to 39.9% by 2060 [2]. A super-aged society faces several problems not only in employment and welfare but also in healthcare. Osteoporosis is a common medical condition occurring in a super-aged society. Recently, Miyoshi et al. reported that the number of patients with osteoporosis is increasing every year and is estimated to be 13 million in Japan [3]. Every year, two million people experience fractures due to osteoporosis, and such fractures affect not only physical functions but also psychological functions causing conditions such as depression [4]. In addition, fragility fractures lead to physical disability, pain, impaired quality of life, increased mortality, and higher healthcare costs [5]. Therefore, treatment as well as preventive countermeasures for fractures should be instituted, as more elderly individuals are expected to experience fractures in the coming decade [6].

According to the World Health Organization (WHO), osteopenia or osteoporosis is defined as a progressive disease of the skeleton characterized by low bone mass and micro-architectural deterioration of bone tissue, resulting in an increased risk of fragility fractures [7]. Osteoporosis is a defect of bone metabolism. Bone remodeling is a process in which old or damaged bone is removed by osteoclasts and replaced with new bone formed by osteoblasts. The bones are maintained and remodeled through the bone remodeling system [8]. Osteoclasts resorb aged or damaged bone, while osteoblasts form new bone. Patients with osteoporosis exhibit an imbalance that leads to increased function of osteoclasts and decreased function of osteoblasts, resulting in bone loss [9]. Osteoblasts and osteoclasts communicate through cell–cell contact and cytokine and extracellular matrix interaction. Osteoblasts can affect osteoclast formation, differentiation, and apoptosis through several pathways, such as the receptor activator of nuclear factor-kappa B (RANK)/RANK ligand (RANKL) pathways [10]. In addition, the remodeling cycle is composed of seven sequential phases, namely quiescence, activation, resorption, reversal, formation, mineralization, and termination, in that order.

The major risk factors for osteoporosis, as defined by the WHO, include aging, low body mass index (BMI), history of fracture, parental history of hip fracture, smoking, alcohol consumption, use of glucocorticoids, and rheumatoid arthritis [7]. Environmental factors include inadequate nutrition, limited physical activity and exposure to sunlight, and risk of falls [7]. Of the many risk factors, aging has been reported as the strongest risk factor, with older women in particular being in the high-risk group [11]. Hormonal involvement has been reported as the leading cause of osteoporosis. Hormones such as estrogen, testosterone, and parathyroid hormone play an important role in bone remodeling by inhibiting bone resorption and promoting bone formation. Therefore, postmenopausal women experience a significant loss of bone mass due to decreased estrogen secretion [7].

On the other hand, the most common causes of tooth loss are dental caries and periodontal disease, with periodontal disease being the most common disease among the elderly population [12]. Periodontal disease triggers an inflammatory response in the periodontal tissues and has local as well as systemic effects through inflammatory cytokines [13,14]. The current model for periodontal disease pathogenesis proposes that oral bacteria aggregate and coexist in a controlled inflammatory state, both the host response and bacterial activity contribute to the bone loss, and inflammation promotes extracellular matrix degradation and bone resorption [14]. In other words, inflammatory cytokines disseminated by periodontal bacteria may affect bone remodeling and be involved in bone formation. Many studies have evaluated the correlation between periodontal disease and bone density. Mohammed et al. conducted a study in 300 postmenopausal women aged 50–70 years to determine the association between systemic osteoporosis and periodontal disease and reported that osteoporosis is a risk factor for periodontal disease and plays an important role in periodontal disease progression [15]. Gil-Montoya et al. performed a study in 173 women and reported that low bone mineral density is associated with high clinical attachment level of gingiva [16]. In addition, Inagaki et al. examined the association of periodontal status and tooth loss with metacarpal bone mineral density in 356 Japanese women, and found that postmenopausal women with fewer than 20 teeth were 1.6 times more likely to have low bone mineral density than those with 20 or more teeth [17]. Periodontal disease and diabetes have been reported to be closely related, which indicates the association between oral health status and bone mineral density [18,19].

According to a study that investigated the differences in osteoporosis prevalence between sexes, men have higher bone mineral density in the hip and higher bone mineral content in the lumbar spine, and women start losing bone at an earlier age and a faster rate than men [20]. Women over the age of 50 years show a four times higher prevalence of osteoporosis, two times higher prevalence of osteopenia, and tend to exhibit bone fractures 5–10 years earlier than men [20]. Hence, sex can be considered a confounding factor that has a significant impact on periodontal disease and bone density. However, previous studies that have assessed this effect had small sample sizes and lack of adjustment for confounding factors.

Therefore, this cross-sectional study aimed to examine the relationship between oral health status and bone mineral density after adjusting for confounders using propensity score analysis in healthy community-dwelling elderly individuals in a Japanese local area.

## 2. Materials and Methods

This study used the data set of some previous studies [21,22]. However, the purpose and statistical analysis are different. The Ethics Committee of the Shimane University Faculty of Medicine approved the study protocol in 2019 (number 4570). Written informed consent was obtained from all participants.

### 2.1. Center for Community-Based Health Research and Education (CoHRE) Study

The Shimane CoHRE cohort study examined the determinants of lifestyle-related diseases, including oral health status, in rural areas in the southern part of the Shimane prefecture (Ohnan-cho), Japan [21,22].

### 2.2. Study Design

This cross-sectional study was performed with the data of participants in the Shimane CoHRE study who were recruited in 2019.

#### 2.2.1. Inclusion Criteria

- Residents covered by the Japan national health insurance
- Residents in Ohnan-cho, Shimane, Japan, aged over 65 years
- Residents who had participated in the 2019 survey

#### 2.2.2. Exclusion Criteria

There were no exclusion criteria.

### 2.3. Collected Data

#### 2.3.1. Background Data

We collected data for the following variables: age (years), sex, BMI (kg/m^2^), and hemoglobin A1c level (HbA1c; %).

#### 2.3.2. Oral Health Status

- Number of Remaining Teeth

A dentist and a dental hygienist conducted dental examinations. The number of remaining teeth was counted, and participants were divided into the following two categories according to the level of oral dysfunction, as defined by the Japanese Society of Gerodontology: participants with less than 20 teeth (Low group) and those with 20 or more teeth (High group) [21,23].

- Number of Untreated Teeth

We classified untreated teeth as those with incipient caries and those with high grade caries, in accordance with the classification by the Japanese Ministry of Health, Labor and Welfare.

- Masticatory Function

Objective masticatory ability was assessed using gummy jelly (a soft chewy candy). The participants were instructed to chew a gummy jelly freely, which was collected after 15 s of chewing, and the number of pieces was counted as described previously [24].

#### 2.3.3. Bone Mineral Density

Bone status was evaluated using quantitative ultrasound (QUS) with Benus α (Ishikawa Seisakusho, Ltd., Ishikawa, Japan). QUS has advantages such as no exposure to radiation, low cost, and portability. QUS enables the evaluation of bone quality, especially the microarchitecture of the calcaneus. The bone mineral density value is estimated in comparison to young adult mean (%YAM) of the same sex as the examinee, that is, 100% means the same bone mineral density as a healthy young man or woman [22].

### 2.4. Statistical Analysis

Since a substantial number of patients had missing data, multiple imputation using an ordinal logistic imputation method was utilized, with the assumption that the missing data were missing at random [25]. The following analyses were stratified by gender in order to eliminate confounding factors. In descriptive statistics, the median (interquartile range: IQR) was calculated. The Mann-Whitney U test was used for the comparison between the Low and High groups divided by 20 teeth as the cutoff value. We used propensity score analyses to balance measurable confounders between Low and High groups. A multivariable logistic regression was used to predict number of remaining teeth based on confounding covariates, including age, BMI, HbA1c, number of untreated teeth, and masticatory function. Each patient was then assigned an estimated propensity score, which was his/her predicted probability of number of remaining teeth on the basis of his/her observed baseline characteristics. Finally, multivariable logistic regressions were also performed by applying propensity scores to adjust for group differences in alternative ways: (1) unadjusted model; (2) multivariable-adjusted model; (3) stratified analysis by within-propensity score quintile; (4) regression adjustment (i.e., inclusion of the propensity score as a linear predictor in the model); (5) propensity score-matching which paired Low and High groups that were similar in terms of their measurable characteristics; and, (6) use of the propensity score to create stabilized weights, defined as the inverse probability of treatment weighting (IPTW) [26,27,28].

All statistical analyses were performed using SPSS version 26.0 software (IBM Japan, Tokyo, Japan). A *p*-value less than 0.05 was considered statistically significant.

## 3. Results

### 3.1. Patients Demographics and Characteristics

In total, 702 participants (306 men and 396 women) were enrolled in the study, and their characteristics are shown in Table 1. The median age was 69.0 (65.0–72.0) years for the whole data, 70.0 (65.0–72.0) years for men, and 69.0 (65.0–72.0) years for women. The median BMI was 22.6 (20.7–24.9) kg/m^2^ for the whole data, 23.0 (21.4–24.9) kg/m^2^ for men, and 22.4 (20.1–24.8) kg/m^2^ for women. The median HbA1c level was 5.9% (5.6–6.2) for the whole data, 5.9% (5.6–6.3) for men, and 5.9% (5.6–6.1) for women. The median number of remaining teeth was 26.0 (19.1–28.0) in the whole data, 26.0 (19.0–28.0) in men, and 26.0 (19.7–28.0) in women. The median number of untreated teeth was 0 (0.0–0.1) in the whole data, 0.0 (0.0–0.0) in men, and 0.0 (0.0–0.3) in women. The median masticatory function was 18.0 (11.0–25.0) for the whole data, 20.0 (11.027.0) for men, and 17.0 (11.0–23.0) for women. The median %YAM was 86.2% (79.1–94.9) in the whole data, 92.0% (84.6–100.0) in men, and 81.6% (76.0–89.8) in women.

### 3.2. Comparison of Each Variable in More than (High Group) or Less than 20 Teeth (Low Group)

Table 2 shows the results of the Mann-Whitney U test for comparisons between the two groups formed according to the number of remaining teeth. In men, the variables significantly differing between the High and Low groups were: age (71.0 (69.0–72.0) years in the Low group, 69.0 (64.0–71.0) years in the High group), HbA1c level (6.1% (5.8–6.4) in the Low group, 5.9% (5.6–6.2) in the High group), and masticatory function (8.0 (3.0–14.7) in the Low group, 24.0 (18.0–28.0) in the High group) (all *p* < 0.01). In women, age (71.0 (68.3–73.0) years in the Low group, 68.0 (63.0–71.0) years in the High group), number of untreated teeth (0.0 (0.0–0.6) in the Low group, 0.0 (0.0–0.0) in the High group), and masticatory function (9.4 (3.2–16.5) in the Low group, 18.0 (13.8–24.0) in the High group) were different between the High and Low groups (all *p* < 0.01). However, %YAM was not significantly different between the High and Low groups in both men and women.

### 3.3. Association between Number of Remaining Teeth and Bone Mineral Density Using Propensity Score Analysis

Table 3 shows the results of the multivariable logistic regression and propensity score analyses. When the analysis was conducted separately for men and women, no significant differences were found in the items for men, but significant differences were found in some results of the stratified analysis (odds ratio (95% confidence interval): 1.07 (1.0–1.15), *p* = 0.05) by propensity score analysis and the result of weighting using stabilized IPTW (odds ratio (95% confidence interval): 27.7 (1.86–414.9), *p* = 0.02) for women.

## 4. Discussion

Our study was conducted in the regional cities of Japan, especially in areas with a super-aged society. Of these, the Izumo City, where the survey was conducted, has a particularly advanced aging population, even more than the national average [29]. Individuals in the median age are defined as early elderly individuals in Japan, and they are relatively young among the elderly population [30]. With regard to BMI as a criterion for evaluating body shape, the target population including both men and women was of the standard body shape category (normal weight: BMI greater than or equal to 18.5 to 24.9 kg/m^2^) when evaluated based on the WHO classification [31]. The value of HbA1c ≥ 6.1% is defined as a criterion for diabetes by the Japanese Diabetes Association, and the study population was unlikely to include patients with diabetes [32]. The number of remaining teeth does not always coincide when comparing self-reported methods and examinations by dental professionals; as the data in this study were obtained from examinations by dentists and dental hygienists, it can be considered reliable [33]. According to the report by Matsui et al., the average number of remaining teeth in the age group of the participants in this study is approximately 24.2–24.9 [33]. However, participants in this study had a larger number of remaining teeth. As for masticatory function, participants of this study had high occlusal status and masticatory function, a finding corresponding to the result of our previous study [24]. Therefore, the target population of this study can be considered a group of healthy elderly individuals who visit dental clinics for routine dental checkups. Hence, the results of this study can be considered generalizable to healthy elderly individuals living in rural areas.

Among men aged 65 to 79 years, older men, current smokers, ex-smokers, and men with a low education level have a significantly higher risk of having 19 or fewer teeth [34]. Additionally, older individuals experience more problems in maintaining oral hygiene and oral health [35]. This study also found a significant difference in age between the High and Low groups, which is consistent with the results of previous studies. Patients with 19 or fewer teeth are more likely to have a high HbA1c level [34,36]. Furthermore, the fewer the number of remaining teeth, the lower the masticatory function; hence, the results of this study can be considered consistent [37]. These results suggest that the target population of this study can be representative of the general population with a common pathology, considering tooth loss due to periodontal disease and the association between diabetes and periodontal disease and between diabetes and bone mineral density [38,39].

Similar to the findings in men, the risk of tooth loss increases with age in women [35,40]. In women, the number of untreated teeth tended to be significantly higher in the Low group. To the best of our knowledge, no literature has assessed this relation. Patients in the Low group had larger number of untreated teeth possibly because they did not visit the dentist regularly. As reported in men, a close relationship was evident between the number of remaining teeth and masticatory function in the women of this study [41,42]. Therefore, when the groups were compared, we found that the target population had pathologies consistent with previous reports, which may increase the validity of this study. At the same time, items that showed significant differences in group comparisons were used as adjustment factors in the subsequent propensity score analysis because of their potential as confounders.

In analyses that adjusted for confounders using propensity score analysis, an association was found between the number of remaining teeth and bone mineral density only in women. As the factor was found to be associated only with the female population, it could be related to the decline in female hormones associated with menopause. The median age of the participants in this study shows that almost all female participants were menopausal. In healthy postmenopausal women, bone resorption factor increases in chronic inflammatory diseases, independent of thyroid hormone treatment, leading to increased bone resorption [43].

During an inflammatory response, cytokines, chemokines, and inflammatory mediators (especially interleukin [IL]-6 and IL-17) alter the expression of RANKL on the surface of osteoblasts [44]. When the expression of RANKL is increased more than that of osteo-protegerin, free RANKL binds to RANK on pre-osteocytes to promote osteoclast formation, leading to bone resorption. In periodontal disease, excessive bone resorption occurs either by upregulation of RANKL or by downregulation of osteo-protegerin, leading to an overall increase in the RANKL ratio, resulting in pathological bone resorption. In addition, tumor necrosis factor-α (TNF-α) directly enhances the expression of RANKL in osteoclasts, promotes osteoclast formation, and enhances the expression of sclerostin in osteoclasts, further promoting osteoclast formation [45]. In fact, experiments in mice have shown that the release of inflammatory molecules such as TNF-α may be responsible for the decline in the quality of bone in the alveolar crest [46]. Experiments in a mouse model simulating osteoporosis due to menopause have shown deterioration of alveolar bone microarchitecture, decreased bone formation rate, and increased osteoclast activity, suggesting that postmenopausal osteoporosis leads to the progression of periodontitis [47]. This has been corroborated by Guiglia et al. who reported that bacteria invading the gingiva may alter the normal homeostasis of bone tissue by direct effects (release of endotoxins from bacteria) and/or indirect inflammatory mechanisms, increasing osteoclast activity and decreasing bone density locally as well as systemically [48].

Menopause-related hormonal changes are known to affect the oral environment, possibly due to changes in the levels of sex hormones, such as estrogen, progesterone, and testosterone, which have an impact on the secretion of proinflammatory cytokines involved in bone resorption [49]. Levin et al. reported that hormone replacement therapy plays an important role in preventing osteoporosis by reducing postmenopausal bone mass loss [50]. In addition, postmenopausal women who undergo estrogen replacement therapy have increased oral bone mass than postmenopausal women who do not [51]. In a report that strongly supports our research, Chang et al. evaluated the association between bone mineral density and tooth loss, including related physiological factors, in 3992 postmenopausal women aged 50 years or older. They found that female bone mineral density and related physiological factors showed a significant relationship with the number of remaining teeth, suggesting that osteoporosis is a risk factor for tooth loss in postmenopausal women [52]. The changes in hormonal balance may explain the absence of any association in men. Therefore, our results are positively supported by both basic and clinical research.

Taking the current evidence into consideration, screening for evaluation of aggravating factors of periodontal disease by bone density assessment should be implemented in clinical practice for women approaching menopause; however, this has not been considered in dental practice. As a future study, a clinical trial in which a screening test using bone densitometry is added during the evaluation of periodontal disease should be undertaken. Nonetheless, oral health care by dentists/dental hygienists, which is a variable factor, may be more important than bone density loss caused by physiological factors, because tooth loss may also be a factor associated with decrease in bone density, especially in elderly female populations. Hence, early intervention for periodontal disease may limit the decrease in bone density [53,54].

This study has three limitations. First, this study did not adjust for confounding factors such as use of bone modifying drugs including bisphosphonates and molecular targeted drugs, hormone use, menopausal status, and reasons for tooth loss. While bisphosphonates are effective in improving bone mineral density, they are also an important regulator as they are associated with periodontal disease, causing bisphosphonate-related osteonecrosis of the jaw and decreasing oral health status [55,56]. Second, since bone density was measured by indirect and alternate methods, it may not accurately reflect the presence or absence of osteoporosis. Third, this was a cross-sectional study, so the causal relationship is not clear. A longitudinal study is needed to determine the causal relationship.

## 5. Conclusions

The results of this study suggest that the number of remaining teeth and bone mineral density may be interrelated and that oral health care by dentists/dental hygienists may play an important role in maintaining bone mineral density in elderly women. Women approaching menopause should be assessed to determine the aggravating factors of periodontal disease by bone density assessment in clinical practice.

## Figures and Tables

**Table 1:** Demographic data, overall and by sex.

Variables	Whole Data (*n* = 702)	Men (*n* = 306)	Women (*n* = 396)
Age (years)	69.0 (65.0–72.0)	70.0 (65.0–72.0)	69.0 (65.0–72.0)
Body mass index (kg/m^2^)	22.6 (20.7–24.9)	23.0 (21.4–24.9)	22.4 (20.1–24.8)
Hemoglobin A1c (HbA1c) level (%)	5.9 (5.6–6.2)	5.9 (5.6–6.3)	5.9 (5.6–6.1)
Number of remaining teeth	26.0 (19.1–28.0)	26.0 (19.0–28.0)	26.0 (19.7–28.0)
Number of untreated teeth	0.0 (0.0–0.1)	0.0 (0.0–0.0)	0.0 (0.0–0.3)
Masticatory function	18.0 (11.0–25.0)	20.0 (11.0–27.0)	17.0 (11.0–23.0)
Bone mineral density (young adult mean, %)	86.2 (79.1–94.9)	92.0 (84.6–100.0)	81.6 (76.0–89.8)

Values are presented as median (interquartile range).

**Table 2:** Comparison of each variable between the High and Low groups.

Variables	Men			Women		
	Low (*n* = 81)	High (*n* = 225)	*p*-Value	Low (*n* = 100)	High (*n* = 296)	*p*-Value
Age (years)	71.0 (69.0–72.0)	69.0 (64.0–71.0)	<0.01 *	71.0 (68.3–73.0)	68.0 (63.0–71.0)	<0.01 *
Body mass index (kg/m^2^)	23.1 (21.7–25.0)	22.9 (21.3–25.0)	0.59	22.1 (19.7–25.1)	22.4 (20.1–24.7)	0.61
HbA1c level (%)	6.1 (5.8–6.4)	5.9 (5.6–6.2)	<0.01 *	5.9 (5.6–6.2)	5.9 (5.6–6.1)	0.36
Number of untreated teeth	0.0 (0.0–0.2)	0.0 (0.0–0.0)	0.24	0.0 (0.0–0.6)	0.0 (0.0–0.0)	<0.01 *
Masticatory function	8.0 (3.0–14.7)	24.0 (18.0–28.0)	<0.01 *	9.4 (3.2–16.5)	18.0 (13.8–24.0)	<0.01 *
Bone mineral density (Young adult mean, %)	91.4 (82.7–98.8)	92.0 (85.8–100.0)	0.61	80.1 (74.8–89.4)	81.7 (76.8–89.8)	0.23

Values are presented as median (interquartile range). *: *p* < 0.05

**Table 3:** Odds ratios for each propensity score analysis to determine the association between number of remaining teeth and bone mineral density.

Variables	Men		Women	
	Odds Ratio (95% CI ^1^)	*p*-Value	Odds Ratio (95% CI ^1^)	*p*-Value
Unadjusted Model	1.002 (0.98–1.02)	0.82	1.01 (0.99–1.04)	0.20
Multivariable-adjusted model	0.98 (0.95–1.004)	0.09	0.99 (0.97–1.02)	0.57
Propensity score-adjusted model				
Stratification	0.98 (0.91–1.06)	0.55	1.02 (0.91–1.15)	0.48
Within-propensity score quintile				
1 (Lowest propensity)	1.003 (0.89–1.13)	0.97	1.04 (0.73–1.49)	0.84
2	0.99 (0.93–1.06)	0.88	1.07 (1.0–1.15)	0.05 *
3	0.96 (0.88–1.04)	0.32	1.01 (0.95–1.08)	0.71
4	0.97 (0.91–1.04)	0.41	0.98 (0.94–1.01)	0.21
5 (Highest propensity)	0.97 (0.93–1.01)	0.19	0.99 (0.95–1.03)	0.61
Regression adjustment	0.98 (0.95–1.004)	0.09	0.99 (0.97–1.02)	0.57
Weighting (stabilized inverse probability of treatment weighting (IPTW))	0.34 (0.00005–23768.9)	0.85	27.7 (1.86–414.9)	0.02 *
Matching 1:1	- ^2^	- ^2^	- ^2^	- ^2^

^1^ Confidence interval. ^2^ When the caliper was set to 0.06 (multiplying the standard deviation of the propensity score (0.29) by 0.2) for men and 0.05 (multiplying the standard deviation of the propensity score (0.23) by 0.2) for women in propensity score matching, no matching data were generated. *: *p* < 0.05.
